# Growth rate-associated transcriptome reorganization in response to genomic, environmental, and evolutionary interruptions

**DOI:** 10.3389/fmicb.2023.1145673

**Published:** 2023-03-22

**Authors:** Yuichiro Matsui, Motoki Nagai, Bei-Wen Ying

**Affiliations:** School of Life and Environmental Sciences, University of Tsukuba, Tsukuba, Ibaraki, Japan

**Keywords:** negative epistasis, transcriptome reorganization, gene network, chromosomal periodicity, genome reduction, environmental stress, experimental evolution

## Abstract

The genomic, environmental, and evolutionary interruptions caused the changes in bacterial growth, which were stringently associated with changes in gene expression. The growth and gene expression changes remained unclear in response to these interruptions that occurred combinative. As a pilot study, whether and how bacterial growth was affected by the individual and dual interruptions of genome reduction, environmental stress, and adaptive evolution were investigated. Growth assay showed that the presence of the environmental stressors, i.e., threonine and chloramphenicol, significantly decreased the growth rate of the wild-type *Escherichia coli*, whereas not that of the reduced genome. It indicated a canceling effect in bacterial growth due to the dual interruption of the genomic and environmental changes. Experimental evolution of the reduced genome released the canceling effect by improving growth fitness. Intriguingly, the transcriptome architecture maintained a homeostatic chromosomal periodicity regardless of the genomic, environmental, and evolutionary interruptions. Negative epistasis in transcriptome reorganization was commonly observed in response to the dual interruptions, which might contribute to the canceling effect. It was supported by the changes in the numbers of differentially expressed genes (DEGs) and the enriched regulons and functions. Gene network analysis newly constructed 11 gene modules, one out of which was correlated to the growth rate. Enrichment of DEGs in these modules successfully categorized them into three types, i.e., conserved, responsive, and epistatic. Taken together, homeostasis in transcriptome architecture was essential to being alive, and it might be attributed to the negative epistasis in transcriptome reorganization and the functional differentiation in gene modules. The present study directly connected bacterial growth fitness with transcriptome reorganization and provided a global view of how microorganisms responded to genomic, environmental, and evolutionary interruptions for survival from wild nature.

## Introduction

Bacterial growth was primarily disturbed by genomic, environmental, and evolutionary interruptions. How the individual interruption contributed to growth fitness was intensively studied in laboratories (Baba et al., 2006; Basan et al., 2020; Kinsler et al., 2020), although these interruptions commonly happened combinative in wild nature. As a representative case of genomic interruption, the genome reduction could cause habitat specialization (Nicks and Rahn-Lee, 2017; Simonsen, 2022), such as increased temperature for thermophiles (Sabath et al., 2013) and decreased pH for acidophiles (Cortez et al., 2022), which was supposed to be the consequence of adaptative evolution in nature (Martinez-Cano et al., 2014; Chu et al., 2021). How the growth fitness of these microbes was influenced by genome reduction and the environmental or evolutionary changes was challenging to analyze in wild nature (Lewis et al., 2021). Instead, genome reduction was conducted in the laboratory by deleting nonessential genomic sequences for bacterial growth (Feher et al., 2007; Kato and Hashimoto, 2007). High-throughput growth assay demonstrated that the genome reduction commonly led to the fitness decrease (Karcagi et al., 2016; Kurokawa et al., 2016), although it might benefit metabolic engineering (Mizoguchi et al., 2007; Morimoto et al., 2008; Vernyik et al., 2020). The decreased fitness could be rapidly improved by experimental evolution (Kawecki et al., 2012; Kurokawa and Ying, 2019), probably owing to the increased mutability of reduced genomes (Nishimura et al., 2017; Lao et al., 2022). The evolutionary improved growth fitness was correlated to the environmental variation (Kurokawa et al., 2022). These findings suggested that the combinations of genomic, environmental, and evolutionary interruptions would cause varied impacts on bacterial growth. The quantitative understanding of the contribution of these interruptions to growth fitness remained largely insufficient.

The gene expression pattern was commonly used to interpret bacterial growth, as it was stringently associated with the exponential growth rate (Scott et al., 2010; Klumpp and Hwa, 2014). Significant correlations of gene expression to growth fitness were reported to a large extent (Klumpp et al., 2009; Wytock and Motter, 2019; Liu et al., 2020), such as the growth rate-correlated gene clusters (Matsumoto et al., 2013) and the environmental adaptation-correlated transcriptomes (Ying et al., 2013; Murakami et al., 2015; Feugeas et al., 2016). Computational analyses observed the theoretical features of the transcriptome commonly, such as the power law (Zipf’s rule) (Ying and Yama, 2018), chromosomal periodicity (Mathelier and Carbone, 2010; Nagai et al., 2020), and epistasis (Ying et al., 2013). Accordingly, there was somehow universality in transcriptome reorganization in response to the genomic, environmental, and evolutionary interruptions. Whether the dual interruption additively accumulated the changes in growth and transcriptome caused by individual interruption was an intriguing question.

The present study employed the *Escherichia coli* (*E. coli*) strains carrying the wild-type, reduced, and evolved genomes. The growth fitness and transcriptome were quantitatively evaluated under regular and environmentally stressed conditions. The growth change and the associated transcriptome reorganization in response to the genomic, environmental, and evolutionary interruptions were analyzed. We tried to illustrate an overview of the growth fitness-associated gene network and discover the working principle participating in transcriptome reorganization responsible for being alive.

## Results

### Canceling effect in bacterial growth

**Figure 1:**
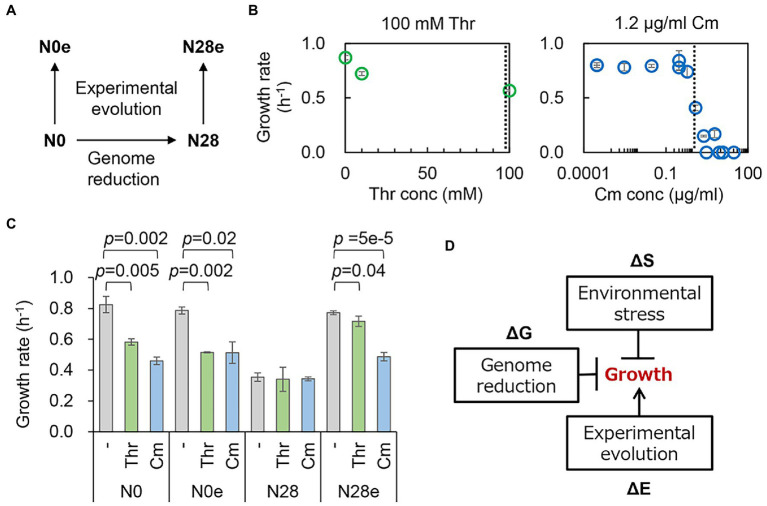
Changes in growth fitness in response to genomic, environmental, and evolutionary changes. **(A)** An overview of the *E. coli* strains. The wild-type and reduced genomes are indicated as N0 and N28, respectively. The evolved N0 and N28 are indicated as N0e and N28e, respectively. **(B)** Contribution of the concentration gradient of the additives to growth fitness. The selected concentrations of the additives are indicated by the broken lines. **(C)** *E. coli* growth rates. N0, N28, N0e, and N28e indicate the wild-type, reduced, evolved, and evolved reduced genomes, respectively. Thr and Cm stand for threonine and chloramphenicol, respectively. The statistical significance (*p* values) of Welch’s *t*-tests are indicated. **(D)** Schematic drawing of the contributions of genomic, environmental, and evolutionary changes to bacterial growth. The changes mediated by genome reduction, environmental stressors, and experimental evolution are represented as ΔG, ΔS, and ΔE, respectively. The arrow and T cross from the boxes stand for the improvement and inhibition of growth, respectively.

The contribution of genome reduction to growth fitness under environmental stress was examined. The wild-type W3110 (N0) and its genome-reduced (N28) strains and their experimentally evolved strains (N0e and N28e) were tested (Figure 1A). The genome size of N28 was approximately 21% smaller than that of N0, and the growth of N28 was significantly slower than that of N0 (Kurokawa et al., 2016). N0e and N28e were experimentally evolved for approximately 1,000 generations in the M63 medium, and N28e showed an improved growth rate compared to N28 (Kurokawa et al., 2022). These strains were the only collection (candidates) well-satisfied with the present study. As the environmental stressors, a number of chemical additives were tested preliminary, i.e., threonine (Thr), chloramphenicol (Cm), NaCl, ampicillin (Amp), and serine (Ser), because they were reported to inhibit the bacterial growth (Bearer and Neet, 1978; Malik et al., 2005; Gutierrez et al., 2013; Matsumoto et al., 2013; Rau et al., 2016). Firstly, the contribution of these additives to the growth of N0 was evaluated in a concentration gradient (Figure 1B; Supplementary Figure S1A). Their concentrations causing the growth rate of N0 to decrease by ~30% compared to that in the regular condition were decided. Secondly, the additives were tested for the growth of N28 at their decided concentrations (Figure 1C; Supplementary Figure S1B). As N28 failed to grow in the presence of NaCl, Amp, and Ser, Thr and Cm were used for the following analyses. Note that the biological function and molecular mechanism of the additives were nonessential for the study; whether they could cause the decreased growth rates of N0 and N28 was crucial.

Repeated growth assays showed that the significant repression in growth was caused by the stressors (Thr and Cm) for the wild-type genome (N0), regardless of the experimental evolution (N0e) (Figure 1C, left). However, the stressor-mediated decrease in growth fitness was undetected in the reduced genome (N28), indicating the reduced responsivity of the reduced genome to the two stressors. Experimental evolution improved the growth fitness of the reduced genome under the regular condition, as previously reported (Choe et al., 2019; Kurokawa et al., 2022), and in the presence of Thr or Cm (Figure 1C, right). It seemed that the evolved N28 (N28e) regained the growth fitness and the responsivity to the stressors. The changes in growth rate indicated a negative epistatic effect in the dual inhibition of growth (Figure 1D). The experimental evolution of the reduced genome released mutual inhibition of growth, i.e., either genome reduction or environmental stress inhibited bacterial growth. Transcriptome analysis was subsequently performed to interpret the canceling effect in growth.

### Homeostatic chromosomal periodicity of transcriptome

**Figure 2:**
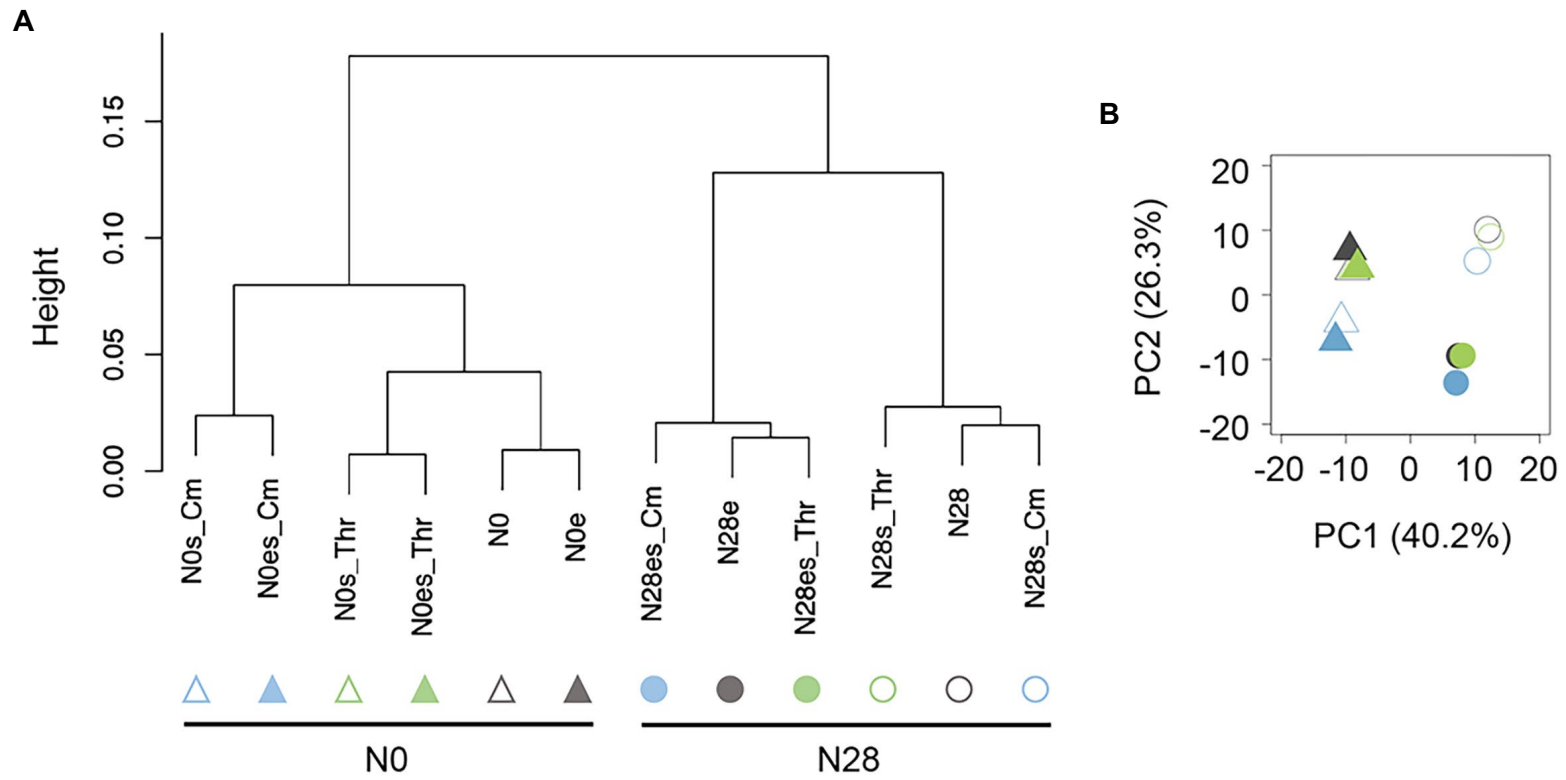
Global gene expression patterns. **(A)** Hierarchical clustering of gene expression. **(B)** Principal component analysis. Triangles and circles represent the wild-type (N0) and reduced (N28) genomes, respectively. Green and blue indicate the addition of Thr and Cm in the culture, respectively. The open and closed signals represent the ancestor and evolved genomes, respectively.

Gene expression patterns were initially analyzed with hierarchical clustering (HC) and principal component analysis (PCA). HC resulted in the primary branch between N0 and N28 (Figure 2A), and PCA showed the most significant differentiation between N0 and N28 at PC1 (Figure 2B). It demonstrated the most significant differentiation in the gene expression pattern was caused by genome reduction. Additionally, the secondary branches were divergent in response to the stressors and experimental evolution in the N0 and N28 clusters, respectively (Figure 2A). It strongly suggested that the wild-type and reduced genomes were highly sensitive to environmental stress and experimental evolution, respectively. These results supported the differentiated changes in the growth fitness of N0 and N28 (Figure 1B).

**Figure 3:**
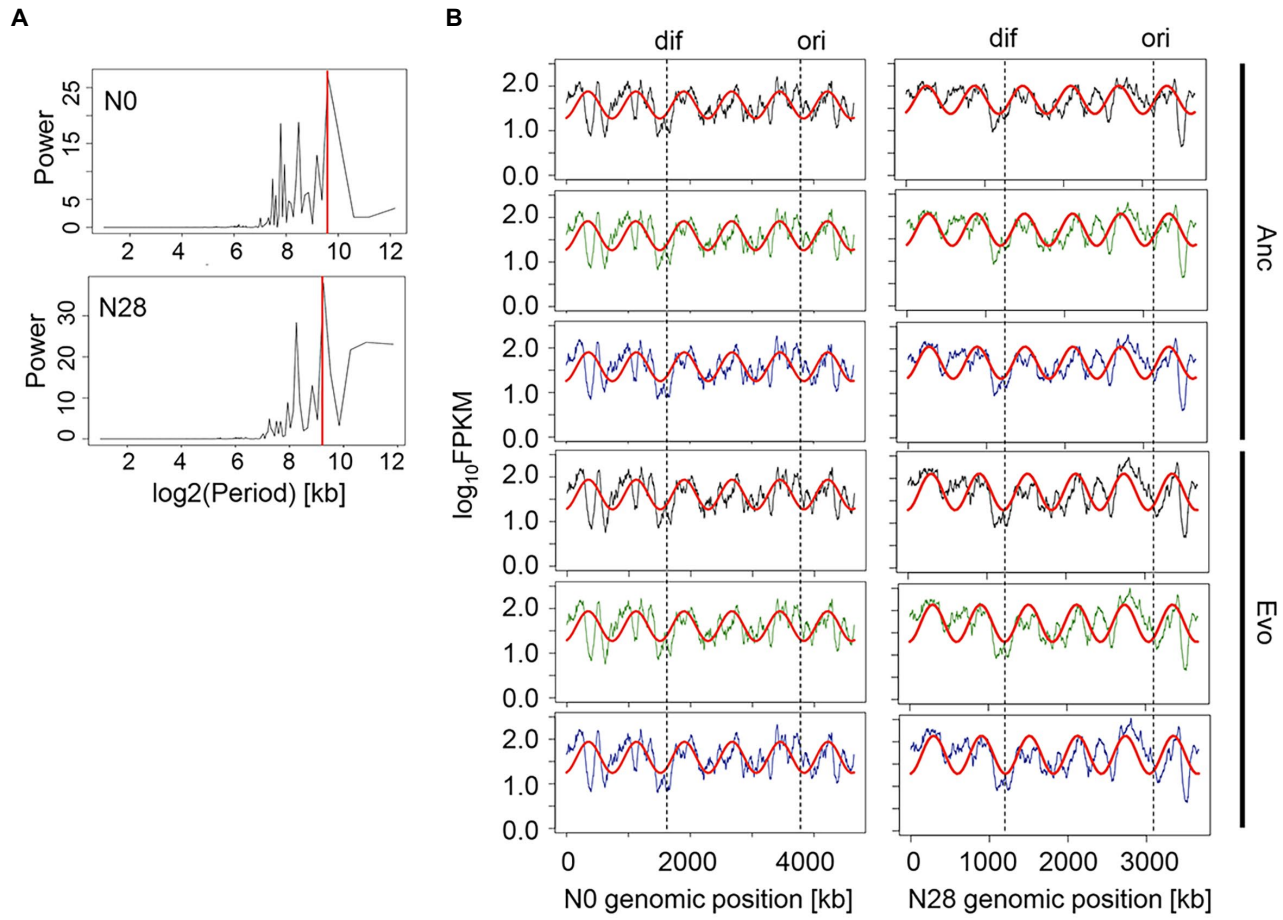
Chromosomal periodicity of transcriptomes. **(A)** Fourier transform. The examples of N0 and N28 are shown. The red lines indicate the highest power spectra (max-peak) estimated by the Fourier transform. **(B)** Chromosomal periodicity. The red curves represent the fitted periods of the transcriptomes according to the max-peaks estimated by the Fourier transform. The transcriptional levels for every 1-kb sliding window and 100-kb smoothing are shown. *Ori* and *dif* are indicated. The left and right panels show the wild-type and reduced genomes, respectively. Anc and Evo represent the ancestral and evolved populations, respectively. Green and blue indicate the addition of Thr and Cm in the culture, respectively.

On the other hand, the global transcriptome reorganization remained homeostatic, despite the differentiation in gene expression patterns due to genome reduction, environmental stress, and experimental evolution. The chromosomal periodicity of the transcriptome was analyzed as previously reported (Nagai et al., 2020). The wavelengths of the maximal power spectra acquired by the Fourier transform of the gene expression were different between N0 and N28 (Figure 3A). The transcriptomes all presented a common six-period of statistical significance (Figure 3B and Supplementary Table S1), consistent with our previous finding (Nagai et al., 2020). The finding first demonstrated that the chromosomal periodicity of the transcriptome was highly conserved, regardless of the genomic, environmental, and evolutionary changes.

### Negative epistasis in transcriptome reorganization

**Figure 4:**
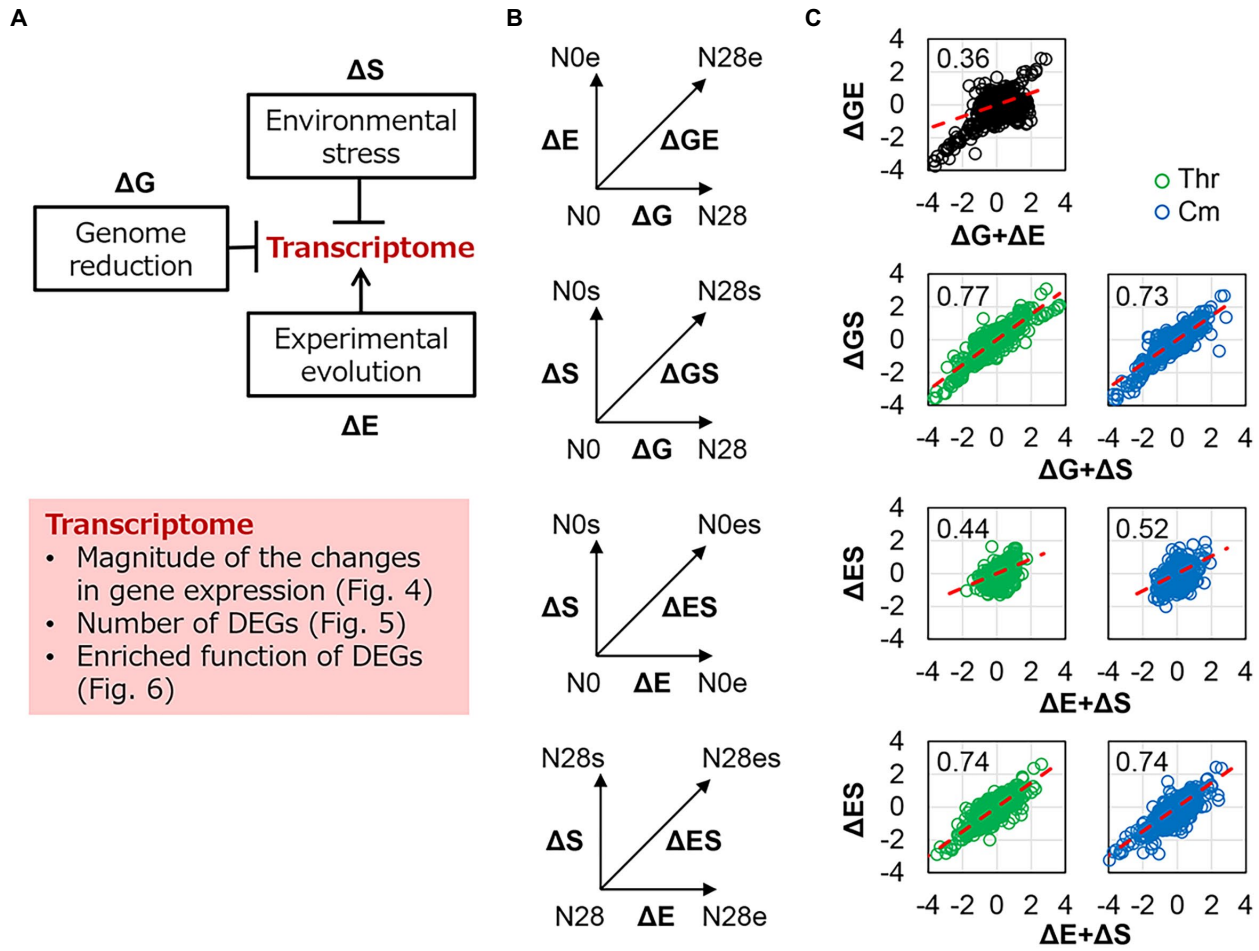
Epistasis in transcriptome reorganization. **(A)** Schematic drawing of the contributions of genomic, environmental, and evolutionary changes to transcriptomes. The box shadowed in pink highlighted the analytical points. **(B)** Schemes of evaluating the epistasis in transcriptome reorganization. ΔG, ΔE, and ΔS represent the transcriptional changes caused by the single interruption of genome reduction, environmental stress, and experimental evolution, respectively. ΔGE, ΔGS, and ΔES represent the simultaneous transcriptional changes caused by the dual interruption of any pairs. **(C)** Epistasis in transcriptome reorganization. The horizontal and vertical axes show the additive changes (the sum of the changes caused by any single interruption) and the simultaneous changes (the changes caused by dual interruptions) in gene expression, respectively. The linear regression slopes are indicated, which were theoretically interpreted in the Materials and Methods. The red dashed lines represent the slope of 1. Green and blue indicate the addition of Thr and Cm in the culture, respectively.

The genetic concept of epistasis was introduced as described previously (Ying et al., 2013). The epistatic change was compared with the additive change, which was evaluated by the sum of individual changes that were different or equivalent to the simultaneous change. In the present study, the changes in transcriptome caused by genome reduction, environmental stress, and experimental evolution were designated as ΔG, ΔS, and ΔE, respectively (Figure 4A), as similar as mentioned in describing the changes in the growth (Figure 1D). The changes in the transcriptome were analyzed in varied viewpoints, i.e., changes in transcriptional levels, differentially expressed genes (DEGs), and enriched regulations and functions (Figure 4A, shadow). Firstly, the transcriptional changes caused by any single genomic (ΔG), environmental (ΔS), and evolutionary (ΔE) interruptions were calculated (Figure 4B, vertical and horizontal arrows). The simultaneous changes caused by any pair of the three interruptions (ΔGE, ΔGS, ΔES) were evaluated as the changes between the two transcriptomes with dual interruptions (Figure 4B, diagonal arrows). The additive changes caused by any pair of the three interruptions (ΔG + ΔE, ΔG + ΔS, ΔE + ΔS) were calculated as the sum of any single changes and were compared to the simultaneous changes by regression (Figure 4C, red lines). The regression slopes varied from 0.36 to 0.77, which were all smaller than 1 (Figure 4C). It demonstrated a negative epistatic effect in transcriptome reorganization triggered by dual interruptions. That is, the changes in gene expression were suppressed by each other of any pair of the three interruptions. For example, the genome reduction (ΔG) somehow canceled out the environmental stress (ΔS) in transcriptome reorganization (Figure 4C, ΔGS). The findings agreed with the insignificant changes in growth fitness of the reduced genome under environmental stress (Figure 1B) and were consistent with our previous finding (Ying et al., 2013). It suggested that the magnitude of the global changes in gene expression was restricted within a certain level, which might benefit the transcriptome homeostasis, such as the conserved chromosomal periodicity (Figure 3).

**Figure 5:**
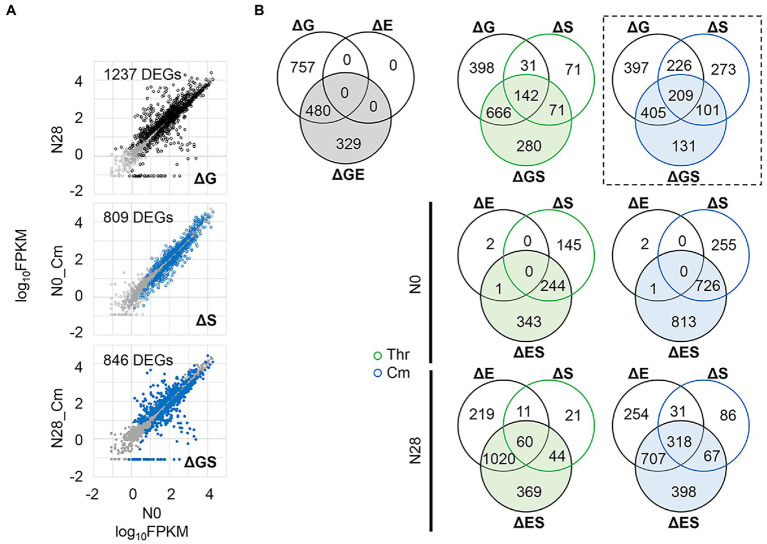
Differentially expressed genes (DEGs). **(A)** An example of identified DEGs. The comparisons of the ΔG-, ΔS-, and ΔGS-mediated changes are shown, in which the number of DEGs is indicated. The DEGs and the other genes are highlighted (in black or blue) and shown in gray, respectively. The gene expression level is shown on a logarithmic scale. **(B)** Venn diagram of DEGs. The numbers of the DEGs mediated by single and dual interruptions are shown. Green and blue indicate the addition of Thr and Cm in the culture, respectively. The box in broken lines indicates the example shown in **A**.

Differentially expressed genes (DEGs) induced by any single or dual interruptions of the genomic, environmental, and evolutionary changes (Figures 4A,B) were determined (Figure 5A and Supplementary Figure S2). For example, 1,237, 809, and 846 DEGs were significantly identified in the ΔG-, ΔS-, and ΔGS-mediated changes in gene expression (Figure 5A), and their overlaps were further analyzed (Figure 5B, box, broken line). Comparing all pairs of DEGs (Supplementary Figure S2) observed that the DEGs were largely overlapped (Figure 5B; Supplementary Figure S3), which strongly supported the epistasis in transcriptome reorganization (Figure 4). ΔS-mediated DEGs largely overlapped with ΔG-mediated DEGs (Figure 5B, ΔGS), with up to as many as approximately 70% overlaps (Supplementary Figure S3). More ΔS-mediated DEGs were detected in N0 and more ΔE-mediated DEGs in N28 (Figure 5B, ΔES). It was consistent with the divergence in growth fitness (Figure 1C) and gene expression pattern (Figure 2) between the wild-type and reduced genomes. There was no overlap between ΔE and ΔS in N0 because there were only three DEGs in ΔE (Figure 5A, ΔGE). Note that the DEGs identified by Rank Product showed similar results (Supplementary Figure S4).

### Gene functions related to the negative epistasis

**Figure 6:**
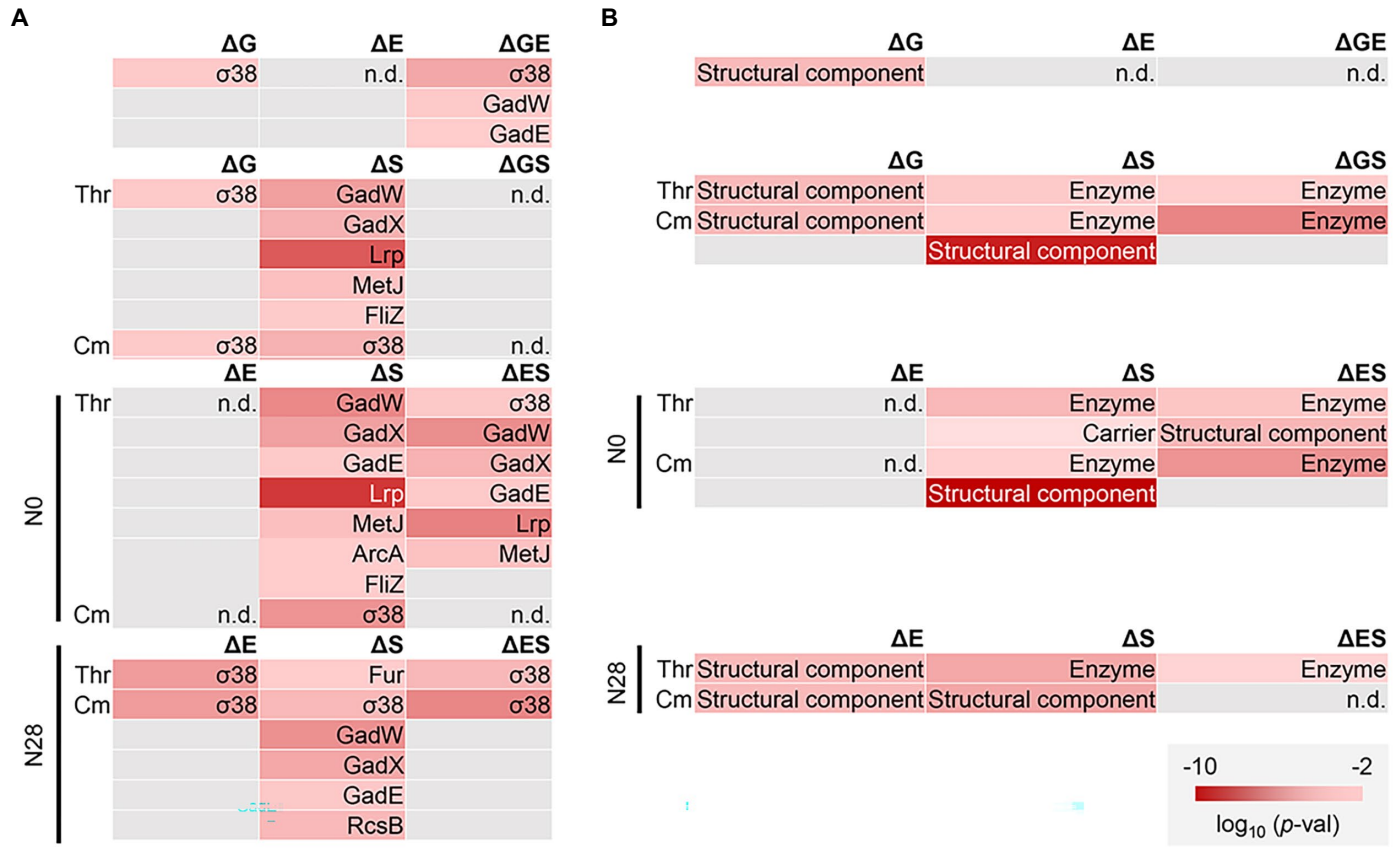
Functional enrichment of DEGs. **(A)** Regulon enrichment. Statistically significant regulons are shown. **(B)** Gene category enrichment. Statistically significant gene categories are shown. Gradation in red indicates the normalized *p* values in the logarithmic scale.

Enrichment analysis was performed to determine the transcriptional regulation and genetic function participating in the transcriptome reorganization. Regulon enrichment of the DEGs showed that a single regulon σ38 and an assortment of regulons, e.g., GadW and GadX, were significant in ΔG and ΔS, respectively (Figure 6A). These regulons were known to be responsible for stress response (Loewen et al., 1998; Tucker et al., 2003). Nevertheless, they were insignificant in ΔGS (Figure 6A, ΔGS). The reduced number of enriched regulons was due to the dual interruption of genome reduction and environmental stress. Such offset tendency was also detected in ΔES (Figure 6A, ΔES). Gene orthology (GO) enrichment of the DEGs showed a reduced number of enriched GO terms due to the dual interruption (Supplementary Figure S5). It strongly supported the negative epistasis in transcriptome reorganization regarding biological processes. Note that the enrichment analysis failed to identify any biological function in the genes of equivalent changes in the additive and simultaneous manners (Figure 4C, upper), as these genes mostly encoded the conserved or predicted proteins (Supplementary Table S5).

Furthermore, the gene categories (GCs) of the structural component and enzyme were significantly enriched, regardless of the genomic, environmental, and evolutionary changes (Figure 6B). The offset tendency of dual interruption was also observed. The enzyme seemed to be a core GC participating in the dual interruptions. Note that the reduced number of DEGs did not result in the reduced number of enriched functions. The results indicated that the negative epistasis occurred in the gene expression and biological function. Additionally, KEGG pathway enrichment of the shared DEGs assigned in the enriched regulons and GCs showed that the amino acid metabolisms played a role in the transcriptome reorganization to a great extent (Supplementary Figure S6).

### Gene modules responsible for the epistatic changes in transcriptome and fitness

**Figure 7:**
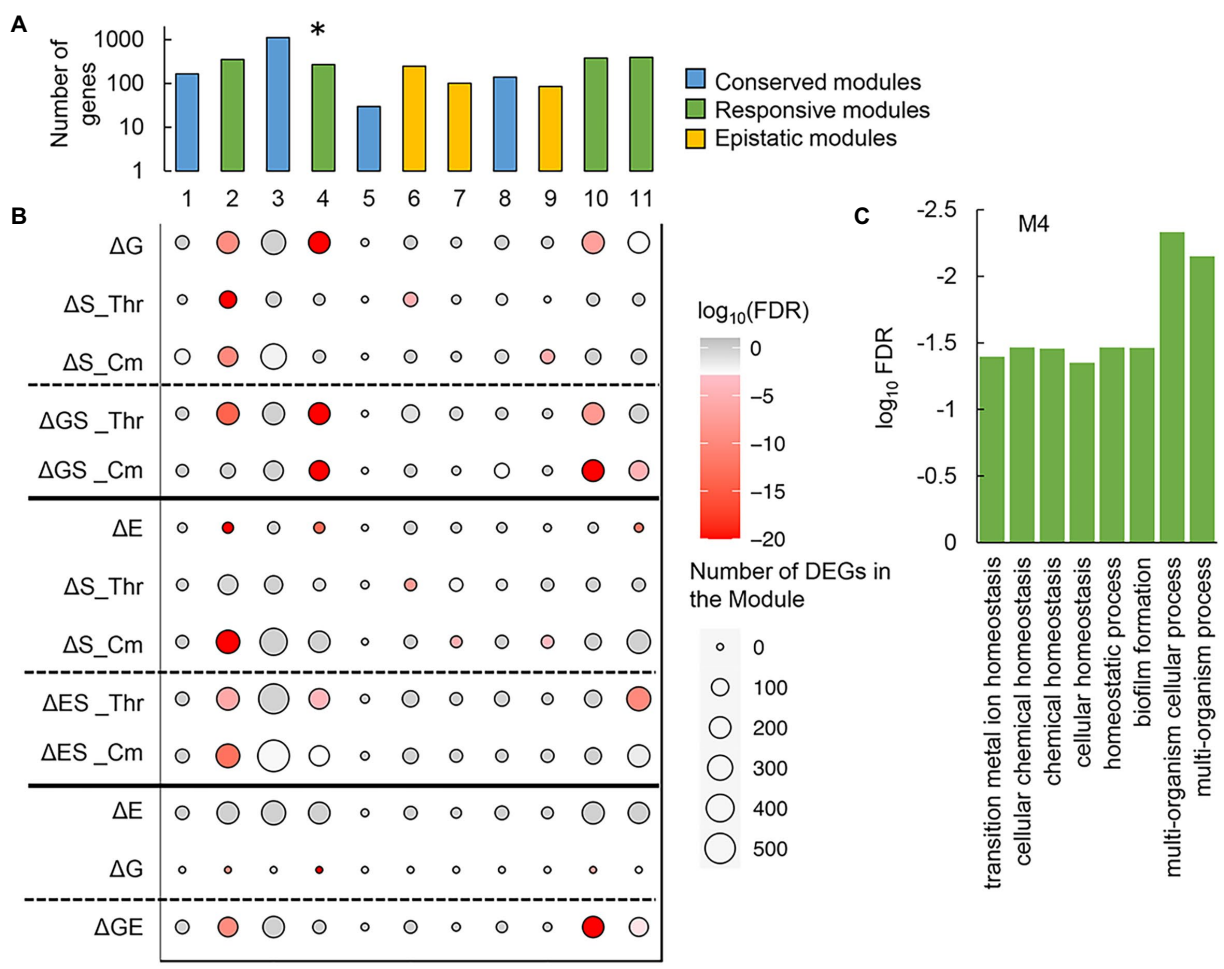
Gene modules clustered by weighted gene co-expression network analysis (WGCNA). **(A)** Gene modules newly constructed by WGCNA. A total of eleven modules newly constructed are shown. Color variation indicates the assigned types of gene modules. The asterisk indicates a significant correlation to the growth rate (*p* < 0.01). **(B)** Enrichment of the DEGs in the gene modules. The numbers of the DEGs mediated by any of the individual and dual interruptions are represented as circles. Gradation in red indicates the statistical significance of enrichment. **(C)** Functional enrichment of M4. The enriched GO terms of high significance (FDR < 0.05) are indicated. Statistical significance is shown in a logarithmic scale.

To figure out the negative epistasis in the transcriptome, novel gene network construction was performed with the weighted gene co-expression network analysis (WGCNA), as described previously (Langfelder and Horvath, 2008; Bhatia et al., 2022). A total of 3,290 genes were classified into 11 modules, which comprised 30 to 1,118 genes per module (Supplementary Figure S7). Functional correlation analysis showed that only one of these modules (M4), which comprised 271 genes (Supplementary Table S3), showed a significant negative correlation to the growth rate (Figure 7A; Supplementary Table S2). Functional enrichment analysis of the genes clustered in M4 showed that the biological processes assigned in the gene orthologs (GO) related to homeostasis were considerably enriched (Figure 7C). It suggested that bacterial growth was stringently associated with functional homeostasis, consistent with the conserved chromosomal periodicity of the transcriptome (Figure 3).

Furthermore, whether the DEGs were significantly enriched in these modules was first evaluated. The modules M1, M3, M5, and M8 (Figure 7A, blue) failed to enrich the DEGs, regardless of the genomic, environmental, and evolutionary changes (Figure 7B). It suggested that these modules were conserved or irresponsible for the genomic, environmental, and evolutionary changes. The DEGs were mainly enriched in the modules M2, M4, M10, and M11 (Figure 7A, green), which revealed that these modules were highly responsive to individual and dual interruptions (Figure 7B). Additionally, the modules M6, M7, and M9 (Figure 7, yellow) specifically enriched the environmental stress-mediated DEGs (ΔS), which turned out to be insignificant once either genome reduction or experimental evolution happened simultaneously (ΔGS, ΔES) (Figure 7B). These three modules were somehow stringently related to the negative epistasis in growth changes and transcriptome reorganization. In summary, the newly constructed gene networks were roughly categorized into three types, i.e., conserved, responsive, and epistatic modules (Figure 7A). Functional enrichment of the epistatic modules found the metabolic process and translation mechanism were significant in M6 and M9, respectively (Supplementary Figure S8). No function was significantly enriched in M7. The varied enrichment results suggested that the negative epistasis in transcriptome reorganization was not based on any specific function but owing to the global coordination. The discriminated functions of gene modules might play a crucial role in maintaining the homeostatic transcriptome architecture for being alive in response to genomic, environmental, and evolutionary changes.

## Discussion

The growth fitness, defined as the growth rate during the exponential phase, was employed to quantitatively evaluate the contribution of the genomic, environmental, and evolutionary changes. The research target in the present study was the steady growth but not the stress response of the *E. coli* strains in the presence of the stressor. To search for the proper stressors, five different additives, i.e., Thr, Cm, NaCl, ampicillin (Amp), and serine (Ser), were initially introduced to the bacterial culture because these additives were reported to reduce the bacterial growth rate (Gutierrez et al., 2013; Matsumoto et al., 2013; Rau et al., 2016). The stressors of Thr and Cm were finally chosen out of the five additives, as they successfully reduced the growth rate but maintained steady (exponential) growth of both strains (Figure 1). As known, Cm was a ribosome-binding inhibitor (Levin et al., 2017), and Thr could lead to isoleucine depletion, consequently, inhibit bacterial growth with a single carbon source (Bearer and Neet, 1978; Hama et al., 1991). They both disturbed the amino acid and protein biosynthesis, which was reported to be correlated to the growth rate of *E. coli* (Basan et al., 2015; Peebo et al., 2015; Aida et al., 2022). The related molecular mechanism might remain functional in the reduced genome N28. On the other hand, adding NaCl, Amp, and Ser failed to cause the reduced growth rate but was somehow lethal to N28 (Supplementary Figure S1). NaCl, Amp, and Ser were known to change the permeability, disturb the cell wall, and cause starvation, respectively (Munita and Arias, 2016; Rau et al., 2016; Yu and Liao, 2018). These stressors might be too severe for the reduced genome, in which the functional genes were deleted or the related molecular mechanisms were inactivated. It might cause a considerably prolonged lag time, which made the exponential growth undetectable within 48 h, the time limit of the temporal growth assay. Thus, these stressors were out of the scope of the present study.

The present study applied the genetic concept of epistasis, initially used to describe the canceling effect caused by multiple mutations in adaptative evolution (Khan et al., 2011; Hartl, 2014), to interpret the canceling effect in the fitness changes responding to the dual interruption of genomic and environmental changes. The canceling effect in response to environmental changes has been found between the heat shock and alcohol stresses (Piper, 1995), and the hydrogen peroxide and ethanol stresses (Semchyshyn, 2014). These findings were known as the cross-stress resistance (Święciło, 2016), describing how the microorganisms acquired strong or multiple resistance. The so-called regulatory cross-talk (Święciło, 2016) was proposed to be the mechanism of cross-stress resistance; that is, a single transcriptional factor responded to various stresses. It might contribute to the negative epistasis observed in the present study. For example, the genes regulated by the transcription factor σ38 were commonly enriched in the ΔG- and ΔS-mediated DEGs (Figure 6A). σ38 was probably responsible for both genome reduction and environmental stress; thus, the growth change was insignificant when the dual interruption occurred (Figure 1B). Taking account of the findings of the enriched functions of translation and ribosome assembly (Figure 6 and Supplementary Figure S8) and the reduced protein synthesis by promoting the formation of 100S ribosome complexes (Trösch and Willmund, 2019), the canceling effect in growth and the negative epistasis might be attributed to the activated σ38 regulon. Note that the canceling effect was based on the fact that N28 growing slower than N0 was independent of the stressors. Although the presence of Cm of Thr could inhibit the ribosomes for translation or cause the depletion of isoleucine, either the abundance of the remaining active ribosomes or the lowered concentration of isoleucine might be sufficient for the slowly growing N28. N28 might benefit from its reduced growth rate. Despite these suspected molecular mechanisms, the negative epistasis in transcriptome reorganization could well explain the canceling effect. It demonstrated that mutual inhibition occurred among the genetic changes (i.e., mutations) and between the genomic and environmental changes.

In addition, to discover the direction of transcriptional changes, the DEGs could be further distinguished between up- and down-regulated by RankProd. Roughly, there seemed to be more downregulated DEGs than upregulated ones in N28 (Supplementary Table S6), indicating the repressed expression of the reduced genome in response to environmental and evolutionary interruptions. Either the genomic (ΔG) or the evolutionary (ΔE) interruption caused more upregulated DEGs, whereas the dual interruptions (ΔGS, ΔGE, ΔES) led to more downregulated DEGs (Supplementary Table S6). Such reversed changes in the number of DEGs supported the epistasis in transcriptome reorganization (Figure 4). The environmental interruption triggered somehow distinguished changes depending on the stressors and genomes. More downregulated DEGs responding to Cm (ΔS_Cm) were commonly found in N0 and N28, whereas more upregulated DEGs responding to Thr (ΔS_Thr) were found in N0 and reversed in N28 (Supplementary Table S6). For instance, dadA, D-amino acid dehydrogenase, was upregulated in N0 (N0, ΔS_Thr); in contrast, no DEGs were related to amino acid metabolism in N28 (N28, ΔS_Thr), indicating that genome reduction reduced the isoleucine suppression by the additional Thr (Supplementary Table S7). The 50S ribosomal subunit proteins, e.g., *rpmD*, *rplO*, and *rplE*, were the upregulated DEGs in N0 (N0, ΔS_Cm); in contrast, no ribosome- or translation-related DEGs in N28 (N28, ΔS_Cm), indicating that genome reduction reduced the inhibition of Cm to 50S ribosome (Supplementary Table S7). The suppression of cellular function by additional Thr or Cm was somehow alleviated by genome reduction. Taken together, considerable changes must have occurred in the amino acid metabolism-, ribosome-, and translation-related functions to achieve the epistatic changes. Further studies on molecular mechanisms are required to clarify the epistatic effect observed in the genomic, environmental, and evolutionary interruptions.

In wild nature, microorganisms constantly face various environmental changes and frequently experience genomic changes, e.g., genome reduction or gene loss, to adapt to their habit (Batut et al., 2014; Giovannoni et al., 2014; Simonsen, 2022). The present study directly connected the genomic, environmental, and evolutionary changes with bacterial growth and transcriptome. Multilevel analyses successfully discovered the negative epistasis in transcriptome reorganization, which was assumed to benefit the homeostasis of transcriptome architecture. Gene network analysis revealed functional differentiation in the gene modules, illustrating an overview of the global optimization of the transcriptome for maintaining growth fitness. These novel findings provided a better understanding of how living organisms responded to the individual and dual interruptions of genome reduction, environmental stress, and adaptive evolution, compensating for known molecular mechanisms and gene functions.

## Materials and methods

### Bacterial strains and culture

The wild-type *E. coli* strain W3110 and the genome-reduced strain (N28) were from the National BioResource Project, National Institute of Genetics, Japan. The reduced genomes were constructed in an accumulated manner, as previously described (Mizoguchi et al., 2008). The evolved *E. coli* strains were adopted from our previous study, in which the strains have undergone experimental evolution in the minimal medium M63 for approximately 1,000 generations (Nishimura et al., 2017). The M63 medium was used for bacterial culture, of which the composition and preparation were described in detail elsewhere (Ishizawa et al., 2015). The chemical compounds, i.e., threonine, serine, NaCl, ampicillin, and chloramphenicol, were commercially available (Wako). Their concentrations in the culture were 100 mM, 75 mM, 0.45 M, 4 μg/mL, and 1.2 μg/mL, respectively, which were determined as the condition where the growth rate of the wild type (N0) decreased by ~30% from the regular culture. The bacterial culture was performed in a 96-well microplate for growth assay and a test tube for RNA sequencing, as described following.

### Growth assay

Every 200 μL of bacterial culture was dispensed to each well of the 96-well microplate (Coaster), as previously described (Kurokawa et al., 2016; Kurokawa and Ying, 2017). In brief, the microplate was incubated at 37°C in a plate reader (EPOCH2, BioTek) shaking at 567 cpm (cycles per minute) for 48 h. The temporal changes in OD_600_ were measured at 30-min intervals. The growth rate was calculated using the following equation between any two consecutive points (Eq. 1)


(1) $$\mu_{i}=\frac{\ln\left(\frac{C_{i+1}}{C_{i}}\right)\;}{t_{i+1}-t_{i}}$$

Here, *t*_i_ and *t*_i + 1_ are the culture times at the two consecutive measurement points, and *C*_i_ and *C*_i + 1_ are the OD_600_ at time points *t*_i_ and *t*_i + 1_. The average of three consecutive μ_i_ showing the largest mean and the smallest variance was determined as the growth rate. The mean of the biological triplicates was defined as the growth fitness and used in the analyses.

### RNA sequencing

Every 5 mL bacterial culture in a test tube was incubated in a bioshaker (BR-23FP, Taitec) at 200 rpm, 37°C. A precision particle size analyzer (Multisizer 4, Beckman Coulter) with an aperture of a pore size of 20 μL was used to evaluate the cell concentration. 20 μL of culture was suspended in a dedicated 25 mL cuvette (Beckman Coulter) containing 10 mL of diluent (Isoton II, Beckman Coulter). The *E. coli* cells were collected during the exponential growth phase (i.e., 5 × 10^7^ ~ 2 × 10^8^ cells/mL). The test tubes were quickly transferred into ice and mixed with Falcon containing 5 mL of a 10% phenol ethanol solution. The Falcon was subsequently centrifuged at 7,000 rpm, 4°C, for 3 min, the supernatant was removed, and the pellet was frozen at −80°C for future use. The pellets were thawed for at least 10 min, and the total RNA was purified using the RNeasy Mini Kit (QIAGEN) and RNase-Free DNase Set (QIAGEN) according to product instructions. Purified total RNA was dissolved in RNase-free water and frozen at −80°C. The rRNAs were removed using the Ribo-Zero Plus rRNA Depletion Kit (Illumina), and the mRNA libraries were prepared using the Ultra Directional RNA Library Prep Kit for Illumina (NEBNext). The paired-end sequencing (150 bp × 2) was performed using the Novaseq6000 next-generation sequencer (Illumina). Biological replications were performed for all conditions (*N* = 2 ~ 6; N0 and N28 under regular conditions were repeated six and four times, respectively; and the others were all duplicates). The raw data sets were deposited in the DDBJ Sequence Read Archive under the accession numbers DRA013430, DRA013683, and DRA015318.

### Data processing and normalization

The reference genome W3110, obtained from GenBank under the accession number AP009048.1, was mapped for the paired-end FASTQ obtained by RNAseq. The mismatch parameter (mp) was set to 2 using the mapping software Bowtie 2 (Langmead and Salzberg, 2012). The obtained read counts were converted to FPKM values according to the gene length and total read count values. Global normalization of the FPKM values was performed to reach an identical mean value in the logarithmic scale in all datasets. The gene expression level was determined as the logarithmic value of FPKM, and the biological replicates were all summarized in Supplementary Table S4. The dataset was used for the following computational analyses.

### Computational analyses

Computational analyses were performed with R. Hierarchical clustering (HC) and principal component analysis (PCA) were performed using the functions of “clusterSample” and “prcomp,” respectively. In HC, the “dist.method” and “hclust.method” were set as “spearman” and “ward.D2,” respectively. In PCA, the “scale” was set as “F.” A total of 3,290 genes common in the wild type and the reduced genomes were used in HC and PCA. Differentially expressed genes (DEGs) were identified using the R package DESeq2 (Love et al., 2014) and the Bioconductor software package RankProd (Breitling et al., 2004; Hong et al., 2006). The read counts and logarithmic FPKM values were used as the input data for DESeq2 and RankProd analyses. The DEGs were determined according to the false discovery rate (FDR) (Storey, 2002). 4,427 and 3,290 genes were used to identify the DEGs for the wild type and the reduced genomes, respectively. In the comparison within the same genome of either N0 or N28 (ΔE, ΔS, and ΔES), either 4,227 or 3,290 genes were used in the analysis (Supplementary Table S6). Once comparing the two genomes of N0 and N28 (ΔG, ΔGS, and ΔGE), the common 3,290 genes in both genomes were used in the analysis.

### Functional enrichment

Functional enrichments of the DEGs were performed according to the features of transcriptional regulation, gene category, gene ontology, and metabolic pathway. A total of 53 transcriptional factors (TFs) (Salgado et al., 2013) comprising more than 10 regulatees and 19 gene categories (GCs) (Riley et al., 2006) of more than 30 genes were subjected to enrichment analysis, as previously described (Ying et al., 2015; Ying and Yama, 2018). A total of 20 GCs were used in the analysis. The statistical significance of the DEGs enriched in TFs and GCs was evaluated by the binomial test with Bonferroni correction. The functional enrichment of gene ontology (GO) (Ashburner et al., 2000; Carbon et al., 2009) and Kyoto encyclopedia of genes and genomes (KEGG) pathway (Kanehisa and Goto, 2000; Kanehisa et al., 2016) analysis was performed using the Database for Annotation Visualization and Integrated Discovery version 6.7 (DAVID) (Huang da et al., 2009). The statistical significance was according to FDR.

### Chromosomal periodicity analysis

A standard Fourier transform was used to evaluate the chromosomal periodicity of the transcriptome, as previously described (Nagai et al., 2020). The periodicity analysis was performed using the function of “periodogram” in the R package. The CDS information was obtained from the DDBJ databank under the accession number AP009048. The periodicity was evaluated with a sliding distance of 1 kb and shown in 100-kb bins of genomic length. The approximate curves of the periodicity were calculated using the max peaks of the periodograms. They were fitted by minimizing the square error of the approximate curves and the series of expression values. The genomic position of *ori* was according to the previous reports (Mizoguchi et al., 2008; Kurokawa et al., 2016). The statistical significance of the periodicity was assessed with Fisher’s g test (Wichert et al., 2004), which was performed using the GeneCycle package in R.

### Epistasis analysis

The epistasis in transcriptome reorganization was performed as described previously (Ying et al., 2013). The transcriptional changes caused by genome reduction or environmental stressors for a given gene were denoted as ΔG or ΔS, respectively. The changes caused by the genome reduction and the environmental stressor simultaneously or separately were defined as ΔGS or ΔG + ΔS, respectively. The linear regression of ΔGS and ΔG + ΔS was performed using the least squares method (Eq. 2). The epistasis in transcriptome reorganization was determined according to the slope of α, as follows.


(2) ΔGS=α(ΔG+ΔS)

α = 1, additivity

α > 1, positive epistasis

α < 1, negative epistasis.

### Gene network analysis

The weighted gene co-expression network analysis (WGCNA) was performed with the R package of WGCNA (Langfelder and Horvath, 2008) under the developer’s instruction. A total of 3,290 genes commonly encoded in the N0 and N28 genomes were subjected to the gene network analysis, where the logarithmic FPKM values were used as the input data. The “step-by-step” method was used to determine the parameters for network construction. The soft threshold of the co-expression network clustering was decided by Scale Free Topology Model Fit, where the R^2^ was approximately 0.9. The FPKM values were converted into the topological overlap matrix (TOM). Hierarchical clustering was performed to divide the genes into various co-expression modules with the “dynamic tree cut” method. The gene modules of similar expression profiles were merged according to the module eigengenes using the “mergeCloseModules” function with a height cut of 0.25. The parameters “softPower,” “minModuleSize,” and “MEDissThres” were set at 12, 50, and 0.25, respectively. The other parameters were set as default. A total of 3,290 genes common in the wild type and reduced genomes were used to construct the network. The statistical significance of the DEGs enriched in the merged modules was evaluated by the binomial test with Bonferroni correction as described above.

## Data availability statement

The data presented in the study are deposited in the DDBJ Sequence Read Archive, accession numbers DRA013430, DRA013683, and DRA015318.

## Author contributions

YM performed the experiments and draft the manuscript. YM, MN, and B-WY analyzed the data. B-WY conceived the research and rewrote the manuscript. All authors approved the final manuscript.

## Funding

This work was supported by the JSPS KAKENHI Grant-in-Aid for Scientific Research (B) (grant number 19H03215) and partially by Grant-in-Aid for Challenging Exploratory Research (grant number 21 K19815).

## Conflict of interest

The authors declare that the research was conducted in the absence of any commercial or financial relationships that could be construed as a potential conflict of interest.

## Publisher’s note

All claims expressed in this article are solely those of the authors and do not necessarily represent those of their affiliated organizations, or those of the publisher, the editors and the reviewers. Any product that may be evaluated in this article, or claim that may be made by its manufacturer, is not guaranteed or endorsed by the publisher.
