# Supplementary material for: Growth rate-associated transcriptome reorganization in response to genomic, environmental, and evolutionary interruptions
Source: Front Microbiol. 2023 Mar 22;14:1145673. doi: 10.3389/fmicb.2023.1145673 (PMC10073601; doi:10.3389/fmicb.2023.1145673)
Supplement: Supplementary file 6 [file Data_Sheet_1.pdf]

Supplementary Information

Growth rate-associated transcriptome reorganization in response to genomic, environmental, and evolutionary interruptions

Yuichiro Matsui[1], Motoki Nagai[1], Bei-Wen Ying[1,*]

[1]School of Life and Environmental Sciences, University of Tsukuba, Tennodai 1-1-1, Tsukuba, Ibaraki 305-8572, Japan

[*]Correspondence: ying.beiwen.gf@u.tsukuba.ac.jp

Supplementary figures and figure legends (Figs. S1-S8) **pp. 2-9**

Supplementary tables (Tables S1, S2) **pp. 10-11**

Supplementary table captions (Tables S3-S7) **pp. 12**

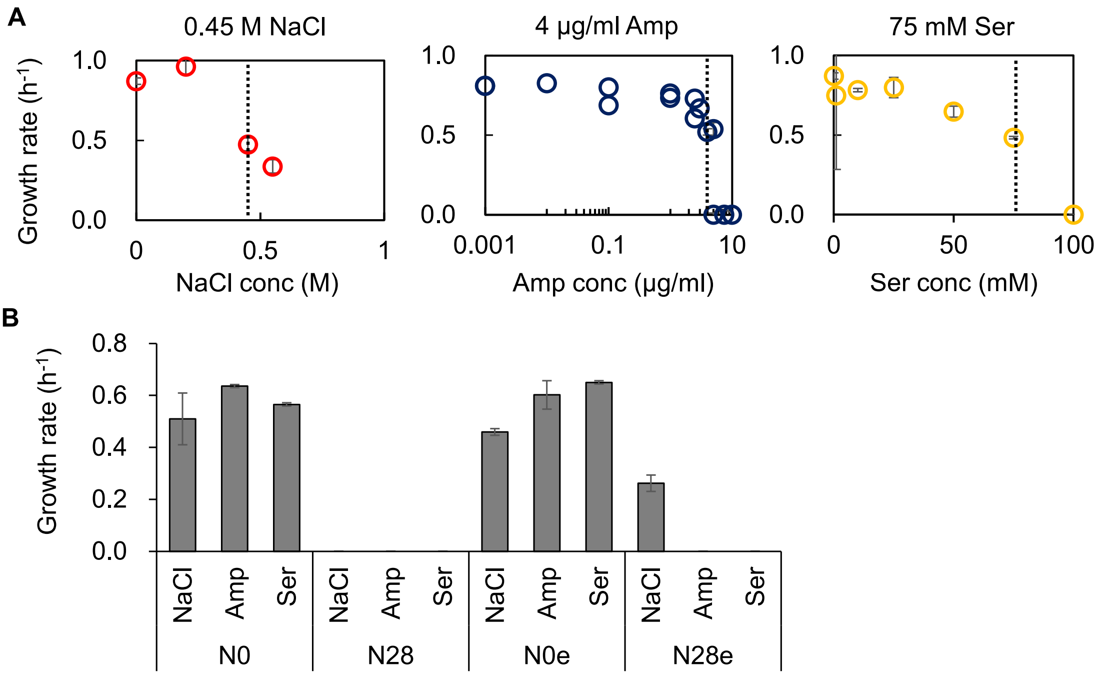

Figure S1 Contribution of other additives to bacterial growth. A. Contribution of the concentration gradient of the additives to growth fitness. Vertical dotted lines indicate the selected concentrations of the additives. **B.** *E. coli* growth rates. N0, N28, N0e, and N28e indicate the wild-type, reduced, evolved, and evolved reduced genomes, respectively. NaCl, Amp, and Ser stand for salt, ampicillin, and serine, respectively.

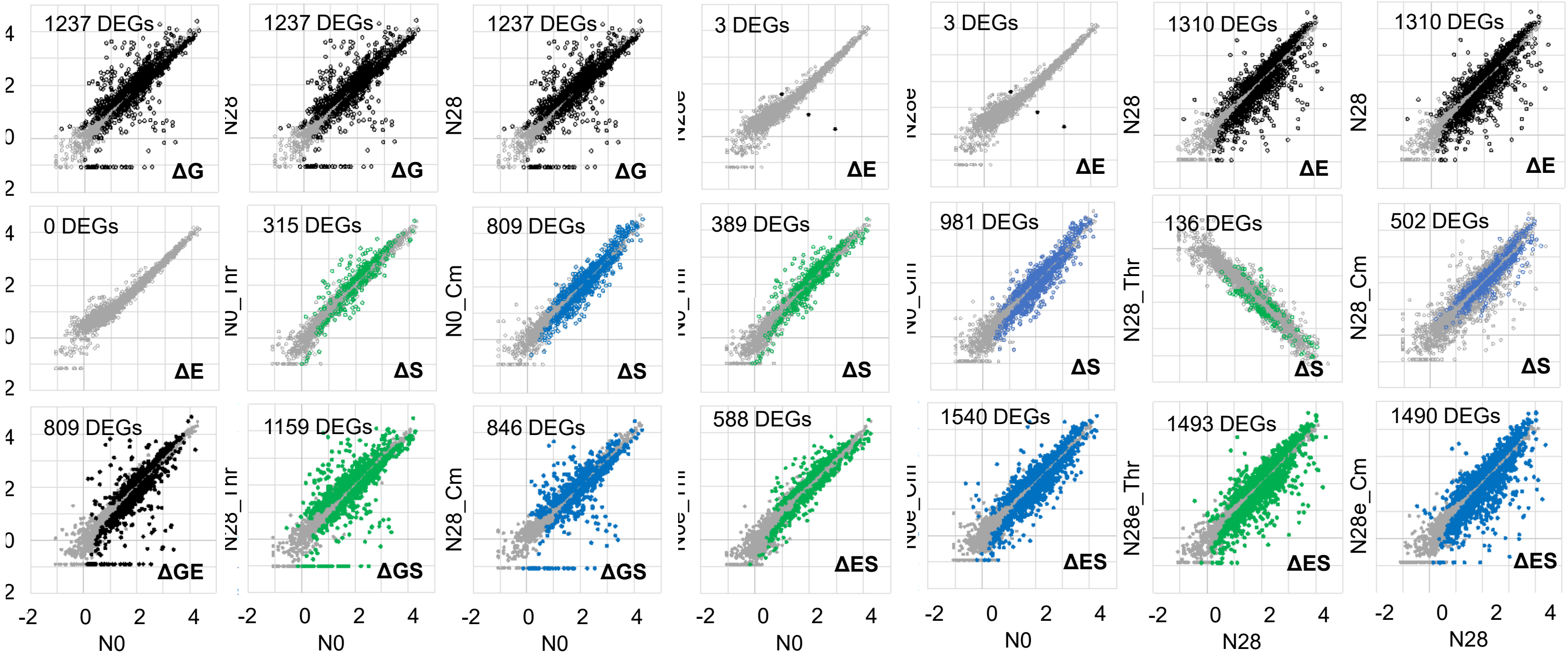

Figure S2 DEGs mediated by genomic, environmental, and evolutionary interruptions. The comparisons of ΔG-, ΔS-, ΔE-, ΔGS-, ΔES-, and ΔGE-mediated changes are shown, in which the number of DEGs is indicated. The DEGs and the other genes are highlighted (in black, green, or blue) and shown in grey, respectively. The gene expression level is shown on a logarithmic scale.

.

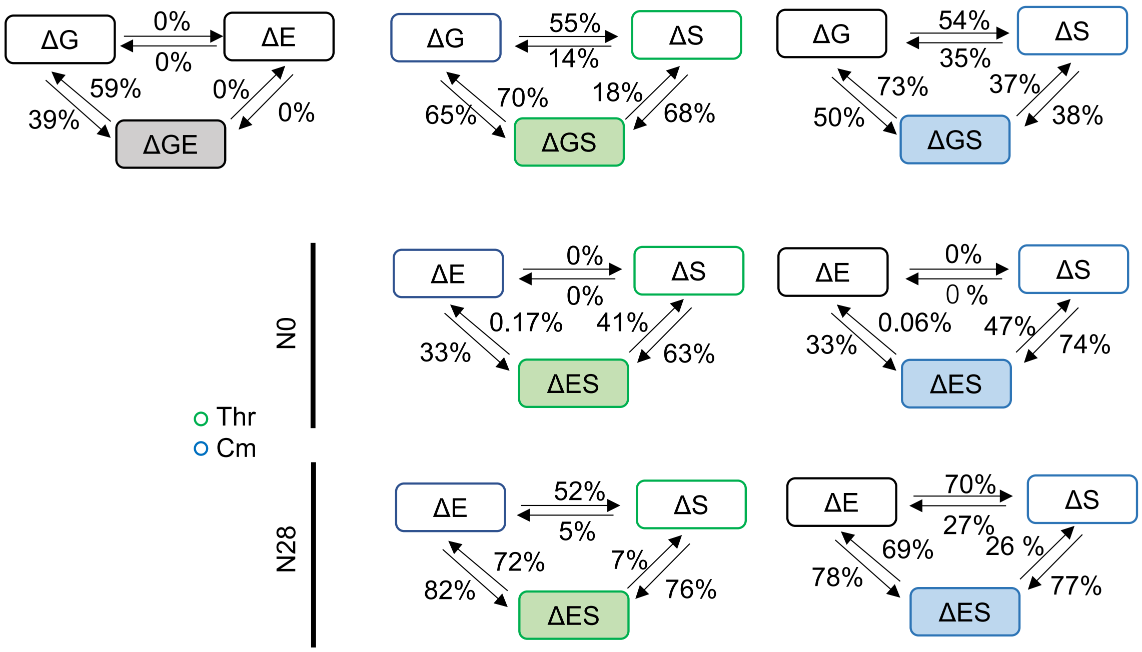

Figure S3 Ratio of overlapped DEGs. Ratios of the common DEGs were calculated according to Fig. 4. The arrows indicate the direction of the division.

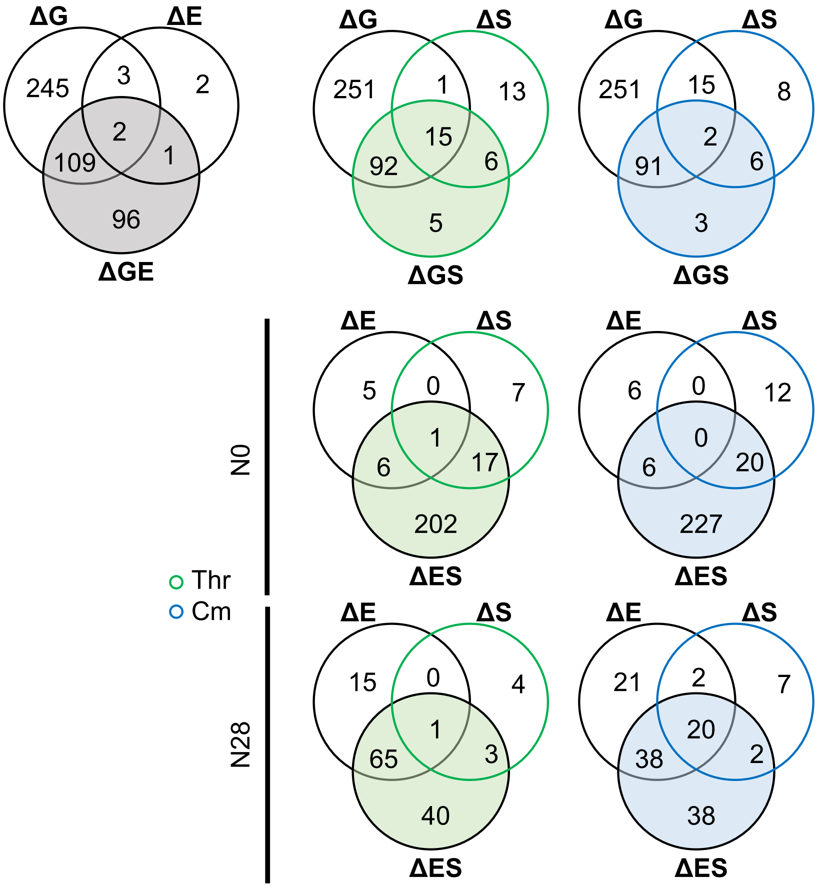

Figure S4 Venn diagram of the DEGs determined by RankProd. The numbers of the DEGs mediated by any of the individual and dual interruptions are shown. Green and blue indicate the addition of Thr and Cm in the culture, respectively.

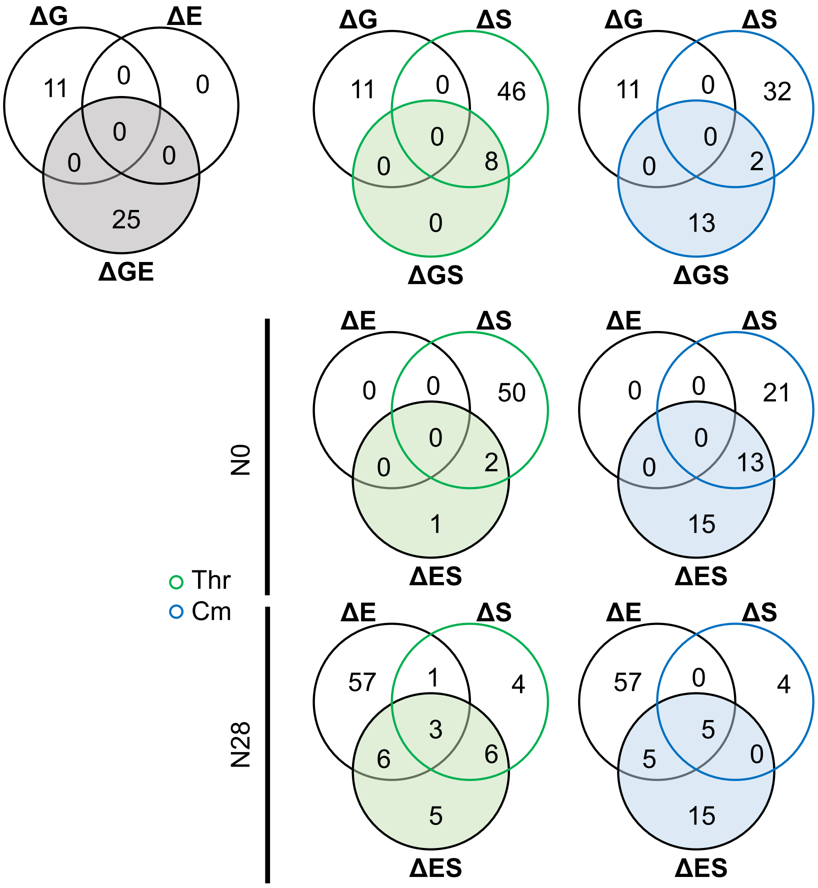

Figure S5 Venn diagram of the GO enrichment of the DEGs. GO enrichment of the DEGs due to individual and dual interruptions was performed. The numbers of significantly enriched GO terms are shown. Green and blue indicate the addition of Thr and Cm in the culture, respectively.

	ΔG	ΔE	ΔGE
	n.d.	n.d.	n.d.

	ΔG	ΔS	ΔGS
Thr	n.d.	Cysteine and methionine metabolism	n.d.
		Glycine, serine and threonine metabolism	
		Biosynthesis of secondary metabolites	
		Biosynthesis of amino acids	
		Metabolic pathways	
		Butanoate metabolism	
Cm	n.d.	Biosynthesis of secondary metabolites	n.d.
		Microbial metabolism in diverse environments	
		Starch and sucrose metabolism	
		Lysine degradation	
		Carbon metabolism	
		Arginine and proline metabolism	

		ΔE	ΔS	ΔES
N0	Thr	n.d.	Biosynthesis of amino acids	Metabolic pathways
			Metabolic pathways	Biosynthesis of secondary metabolites
			Cysteine and methionine metabolism	Biosynthesis of amino acids
			Glycine, serine and threonine metabolism	Valine, leucine and isoleucine biosynthesis
			Lysine biosynthesis	Butanoate metabolism
			Biosynthesis of secondary metabolites	Alanine, aspartate and glutamate metabolism
				Cysteine and methionine metabolism
				Pantothenate and CoA biosynthesis
				Arginine and proline metabolism
				2-Oxocarboxylic acid metabolism
	Cm	n.d.	Biosynthesis of secondary metabolites	n.d.
			Microbial metabolism in diverse environments	
			Starch and sucrose metabolism	
			Lysine degradation	
			Carbon metabolism	
			Metabolic pathways	
N28	Thr	n.d.	Biosynthesis of siderophore group nonribosomal peptides	Arginine and proline metabolism
			Biosynthesis of secondary metabolites	Metabolic pathways
				Biosynthesis of secondary metabolites
				Carbon metabolism
	Cm	n.d.	Quorum sensing	n.d.
			Biosynthesis of secondary metabolites	

Figure S6 Enriched KEGG pathways. The common genes in the enriched gene category and regulons were subjected to the enrichment analysis of KEGG pathways. Statistically significant pathways are shown. Gradation in red indicates the normalized p values in the logarithmic scale.

A

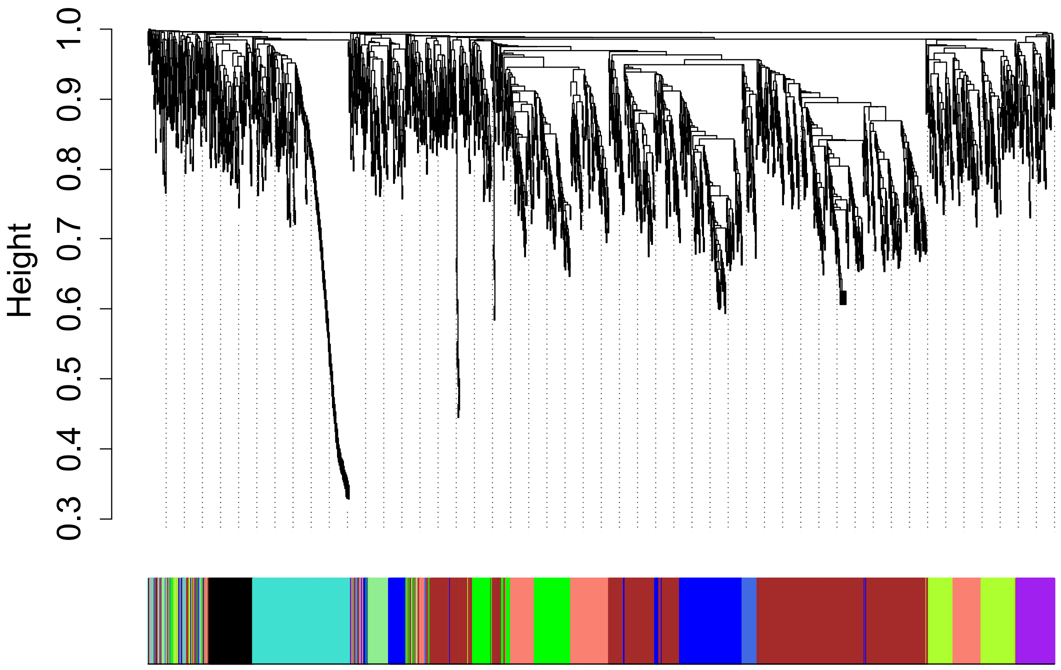

B

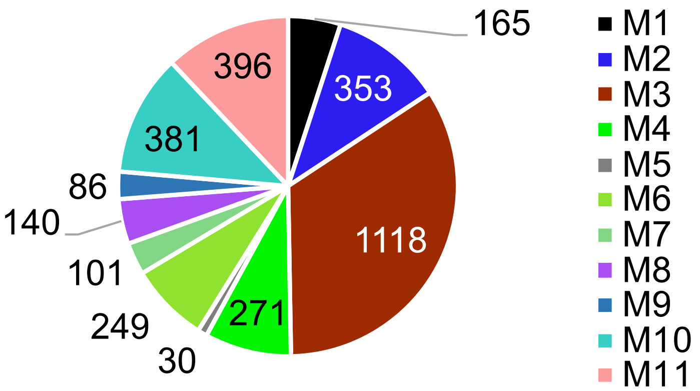

Figure S7 Weighted gene co-expression network analysis (WGCNA). A. Hierarchical clustering of the gene network. **B.** Pie chart of the numbers of the genes assigned in each module. Color variation indicates the eleven gene modules.

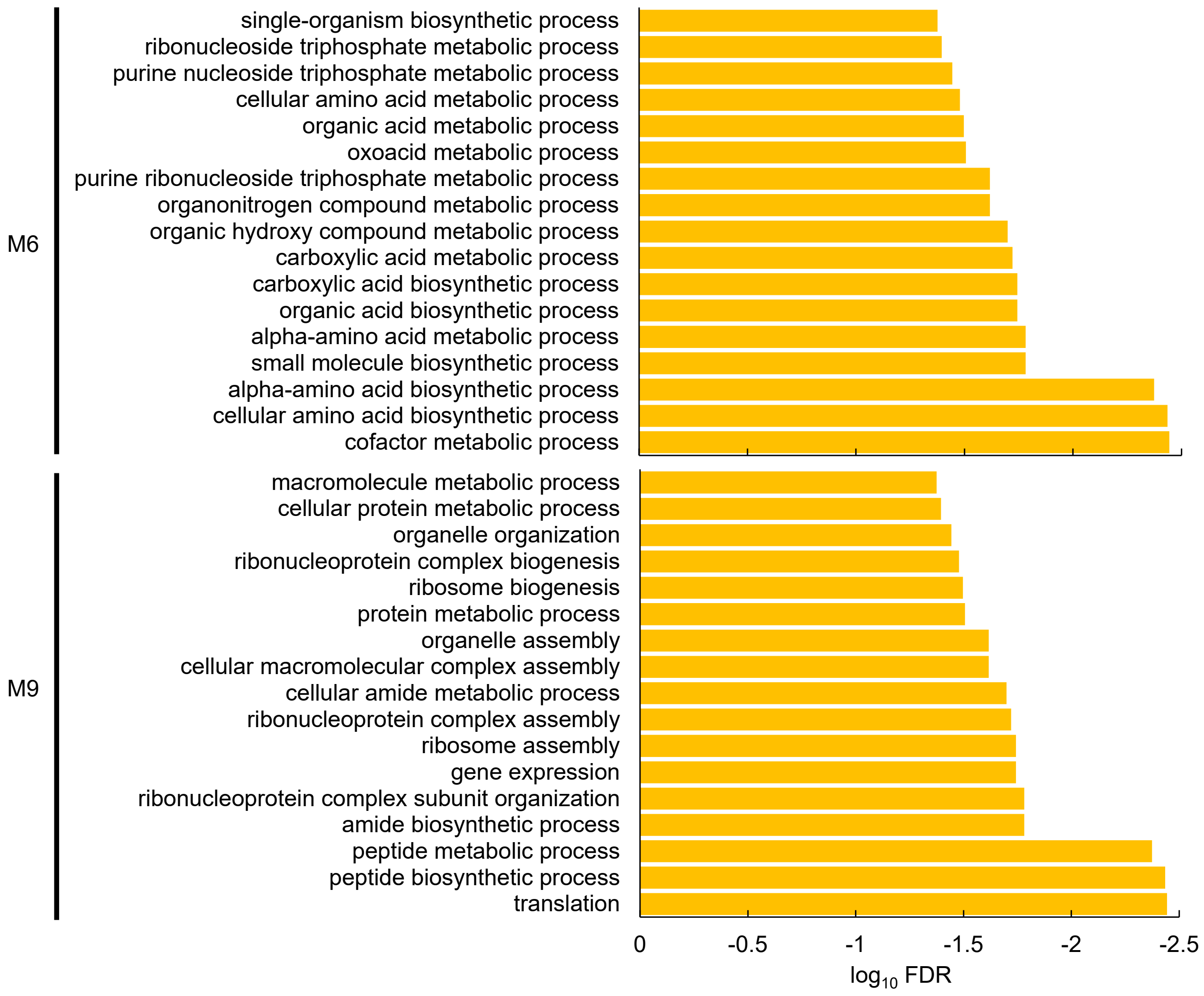

Figure S8 Functional enrichment of the epistatic modules. The upper and bottom panels indicate the enrichment of the genes in modules of M6 and M9, respectively. The enriched GO terms of high significance (FDR < 0.05) are indicated. Statistical significance is shown in a logarithmic scale.

Table S1 Chromosomal periodicity analysis of the transcriptomes. Max peak, Fisher-g, and *p* indicate the most significant wavelength obtained according to the highest power spectra in the Fourier transform, the Fisher's g test of the period, and the statistical significance, respectively. NA, Thr, and Cm represent no additive, threonine, and chloramphenicol in the M63 medium, respectively.

Strain	Additive	Genome size (Kb)	Max peak (Kb)	Periods	Fisher-g	*p*
	NA	4,646	774.3	6	0.13	7e-142
N0	Thr	4,646	774.3	6	0.14	1e-151
	Cm	4,646	774.3	6	0.14	6e-146
	NA	4,646	774.3	6	0.18	1e-199
N0e	Thr	4,646	774.3	6	0.14	2e-150
	Cm	4,646	774.3	6	0.14	5e-154
	NA	3,664	610.7	6	0.16	3e-135
N28	Thr	3,664	610.7	6	0.17	2e-149
	Cm	3,664	610.7	6	0.17	4e-149
	NA	3,664	610.7	6	0.16	6e-137
N28e	Thr	3,664	610.7	6	0.17	1e-146
	Cm	3,664	610.7	6	0.17	2e-144

Table S2 Correlation coefficients of the gene modules to the growth rate. Module, *cor*, and *p* indicate the number of gene modules shown in Fig. S7, correlation coefficients, and statistical significance, respectively.

**Module**	***cor***	***p***
M1	0.134	0.678
M2	-0.392	0.207
M3	0.005	0.987
M4	-0.725	0.008
M5	0.309	0.328
M6	-0.132	0.682
M7	0.089	0.783
M8	-0.458	0.134
M9	-0.169	0.600
M10	-0.307	0.332
M11	0.508	0.092

Table S3 Genes clustered in M4. The gene name, ID, and product of 271 genes in module M4 are indicated.

Table S4 Datasheet of gene expression. The gene name and ID are indicated. The gene expression levels of all biological replicates are shown as the logarithmic FPKM values.

Table S5 Genes of equivalent changes in the additive and simultaneous manners. The genes located close to the diagonal of the additive and simultaneous changes ratio from 0.9 to 1.1 were summarized. The gene name, ID, and production are indicated.

Table S6 Number of the DEGs determined by RankProd. Conditions, All genes, All DEGs, Upregulated and Downregulated represent the comparison pairs, the number of genes subject to the DEGs analysis, the number of identified DEGs, the numbers of upregulated and downregulated DEGs, respectively.

Table S7 Gene list of the DEGs determined by RankProd. The gene ID, name, and product of the DEGs are summarized. Conditions and regulatory direction indicate the comparison pairs and whether the DEGs were up- or down-regulated, respectively.
